# Single-sequence low-field 3D T1-weighted MRI for scaphoid fracture detection compared to radiography: prospective diagnostic accuracy pilot study

**DOI:** 10.1007/s10140-026-02481-3

**Published:** 2026-05-02

**Authors:** Claudia Neubauer, Christopher Schuppert, Carolin S. Reidelbach, Horst Zajonc, Filip Simunovic, Sebastian M. Goerke, Fabian Bamberg, Sebastian Wirtz, Maximilian F. Russe, Thierno D. Diallo, Jakob Neubauer

**Affiliations:** 1 Department of Diagnostic and Interventional Radiology, Medical Center – University of Freiburg, Faculty of Medicine, University of Freiburg, Freiburg im Breisgau, Germany; 2 Department of Hand and Plastic Surgery, Medical Center – University of Freiburg, Faculty of Medicine, University of Freiburg, Freiburg im Breisgau, Germany; 3 Present Address: Stühlingerstr. 24, 79106 Freiburg, Germany; 4 Present Address: Radiology Team, Lahr, Germany

**Keywords:** Carpal trauma, Low-field MRI, Scaphoid fracture, Radiography, Musculoskeletal MRI

## Abstract

**Purpose:**

To determine the diagnostic accuracy of a novel high-resolution 3D T1-weighted gradient-echo sequence in low-field MRI for detecting scaphoid fractures compared with radiography.

**Methods:**

In this prospective single-center study, patients with suspected scaphoid injury underwent wrist radiography and 0.31 T extremity MRI including a 4-min 3D gradient-echo sequence (3D SST1). Three musculoskeletal radiologists independently evaluated radiographs and the isolated 3D sequence in separate sessions, rating fracture presence, displacement/stability, diagnostic confidence (five-point Likert scale), and need for additional imaging. The reference standard was based on CT and standard MRI sequences, evaluated by two experienced musculoskeletal radiologists.

**Results:**

Forty patients with 11 scaphoid fractures were included. Across readers, AUC for scaphoid fracture detection was 1.000 for 3D MRI and 0.823 for radiography (AUC-difference 0.177, 95% CI: -0.004–0.359, *p* = 0.056). For 3D MRI, sensitivity and NPV were 1.000 for all readers. Specificity (0.931–0.966 vs 0.655–0.862) and PPV (0.846–0.917 vs 0.444–0.667) were higher than for radiography. Diagnostic confidence was excellent (median score 1) with near-perfect inter-rater agreement (κ = 0.961). Additional imaging was not recommended in 40/36/37 cases (reader 1/2/3).

**Conclusion:**

A single 4-min high-resolution 3D low-field MRI sequence enabled highly accurate detection of scaphoid fractures with perfect sensitivity and NPV, a high reader confidence, and an improved performance compared to radiography in this small pilot cohort. This approach may facilitate rapid, radiation-free screening in acute wrist trauma.

**Graphical Abstract:**

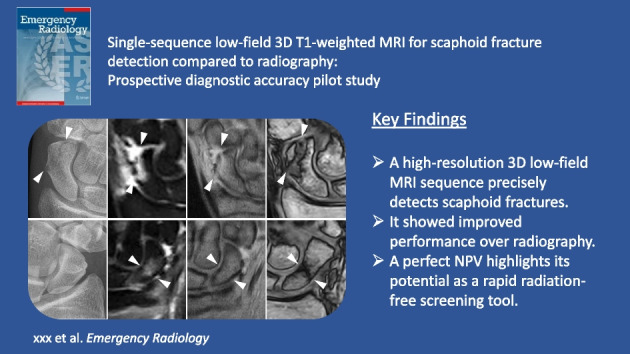

## Background

Approximately 10% of wrist fractures involve the carpal bones. Scaphoid fractures account for roughly 60–70% of carpal fractures [1, 2]. X-ray still serves as the primary diagnostic tool in case of suspected scaphoid fracture despite missing about 30% of acute fractures [2], which may result in a delay of adequate therapy and severe posttraumatic complications [3]. Therefore, multi-slice computed tomography [4, 5] or musculoskeletal cone-beam computed tomography examinations [6–11] of the carpus play an important additional role in the assessment of acute carpal injuries regarding morphology, classification, and stability of fractures [2]. Nonetheless, musculoskeletal MRI represents the gold standard and is highly sensitive for scaphoid fracture detection [2, 12–16]. A 3D T1-weighted GRE sequence on 3TMRIs yielded three-dimensional CT-like images, which, similar to CT, allows beneficial reconstructions in all directions and along the axis of the scaphoid [17, 18], and showed excellent sensitivity, specificity and accuracy for fracture detection of the wrist including the scaphoid [19]. In contrast to X-ray or CT examinations, assessment of the carpal bones using MRI alone would be of particular advantage in younger patients and patients with repeated investigations or follow-ups. It completely avoids radiation exposure and, if in initial or repeated X-ray investigations fractures are not clearly detectable or excluded, delay of treatment or unnecessary immobility [12–16, 19].

The aim of this study was to evaluate the diagnostic accuracy of a single isotropic 3D T1-weighted MRI sequence acquired on a dedicated 0.31-T extremity low-field system for the detection of scaphoid fractures within a short acquisition time. The objective was to determine whether this approach could serve as a high-quality, radiation-free diagnostic option for a predominantly young patient population.

We hypothesized that the 3D MRI sequence would demonstrate diagnostic accuracy comparable to the reference standard for both scaphoid fracture detection and stability classification.

## Methods

Our study was approved by the ethics committee of our institution (approval number 34/16, final approval date 09/08/2017) and conducted in accordance with the Declaration of Helsinki. All patients participating in the study provided written informed consent before participation.

### Patients

Consecutive patients presenting to our institution between May 2018 and April 2021 with suspected traumatic wrist fracture and a clinical indication for wrist CT were eligible for inclusion. Inclusion criteria were: (1) availability of wrist radiographs and carpal CT, and (2) clinical suspicion of a scaphoid fracture, defined by typical symptoms such as dorsal wrist pain and/or tenderness in the anatomical snuffbox.

**Fig. 1:**
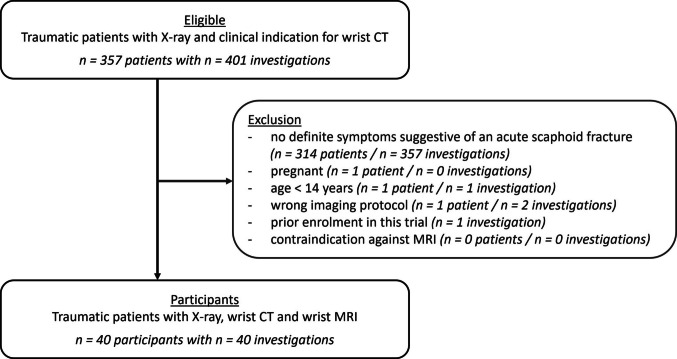
Study flow chart

Exclusion criteria were pregnancy, age under 14 years, prior participation in this study, an incorrect imaging protocol, or contraindications to MR I (Fig. 1).

### MRI examinations

MRI examinations were conducted in all participants using a dedicated extremity 0.31 Tesla MRI system (O-Scan, Esaote, Genoa, Italy; manufactured and installed in 2016) equipped with a specialized DPA hand/wrist coil. The imaging protocol comprised coronal STIR and T1-weighted XBONE sequence, as well as coronal, sagittal, and transverse T2-weighted XBONE sequences. We additionally performed a coronal high-resolution gradient-echo sequence with a special second signal encoding in the direction of the selection gradient (3D SST1). This enables isotropic 3D reconstructions from a volume rather than from individual slices. Parameters of that sequence included an echo time of 12 ms, an extremely short repetition time of 24 ms, radiofrequency spoiling, a low flip angle of 30°, a matrix of 512 × 512 pixels and a field of view of 14 × 14 cm over 6–9 cm. Slice thickness was 0.31 mm with the possibility of performing isotropic reconstructions as necessary for scaphoid evaluation of 0.31 mm. The standard acquisition time of the 3D SST1 sequence was 4:04 min and increased up to 4:35 min depending on the third dimension. Further acquisition parameters are given in Table 1.

**Table 1:** Sequence parameters

	STIR	T2	T1	3D SST1
Acquisition type	2D	2D	2D	3D
Field of view [cm]	14 × 14	14 × 14/14 × 14/12 × 12	14 × 14	14 × 14
Orientation	coronal	coronal/sagittal/axial	coronal	coronal
Matrix size	256 × 256	512 × 512	512 × 512	512 × 512
Pixel spacing	0.625/0.625	0.3125/0.3125	0.3125/0.3125	0.3125/0.3125
Slice thickness [mm]	3	3	3	0.313
Slice spacing [mm]	0.3	0.3	0.3	0.003
Repetition time (TR)	1980*	960/920*/960*	690*	24
Echo time (TE)	25	11/16.5/16.6/22	11/16.5/16.6/22	12
Flip angle	90	35/35/35	45	30
Acquisition time [min:s]	4:45–6:32*	5:31/4:22–6:58/5:35	3:30–4:34*	4:04 (4:24/4:35)

*Adjustments according to patient status

For reference standard all CT-based examinations and all standard MRI sequences except the 3D SST1 and radiography were considered. For evaluation only the 3D SST1 was provided to the readers.

MRIs were preferably performed on the day of X-ray examinations (*n* = 16) with a median interval of 1 day (IQR 0–2).

### X-ray based imaging

36 out of 40 participants received triple-view scaphoid series comprised of a posteroanterior, lateral and angled posteroanterior projection of the carpus in ulnar deviation. In 4 out of 40 cases (3 cases without fracture, 1 case with a scaphoid fracture) an angled posteroanterior projection could not be obtained owing to pain. In addition, all 40 participants received clinically indicated CT examinations of the carpus. Most CT scans were performed on the same day as radiography (median interval 0 days, IQR 0–1).

### Image analysis

The reference standard assessment was performed by two board-certified radiologists, each with 15 years of experience in musculoskeletal radiology, who were blinded to the index test results. The final diagnosis was established using a best available reference standard based on CT, standard MRI sequences, and relevant clinical and follow-up information when available. In cases of disagreement, a joint consensus review was performed.

Scaphoid fractures in the reference standard were diagnosed on the basis of definite fracture findings on combined CT and standard MRI assessment. Follow-up imaging, when available, was used as supportive information but was not required in all patients.

A fracture was classified as displaced if one or more of the following predefined criteria were present: (1) fracture gap > 1 mm, (2) step-off > 1 mm, (3) angular deformity > 15°, (4) translation > 20% of the scaphoid width, (5) evidence of comminution or associated carpal instability.

Fractures not meeting these criteria were classified as non-displaced. In cases of disagreement, a joint consensus review was conducted.

For evaluation of the index tests, three additional board-certified radiologists with 10, 10, and 6 years of experience in musculoskeletal imaging independently assessed the radiographs and the 3D SST1 sequence. These readers were aware of the clinical suspicion of a scaphoid fracture but were blinded to all other clinical and patient information. Radiographs and 3D SST1 datasets were anonymized and presented in random order within the PACS (DeepUnity, version 2.3.0.0, Dedalus Healthcare Systems, Bonn, Germany) in a different order for each modality. There was a time gap of at least 12 weeks between the evaluation of X-rays and the SST1 sequence to minimize recall bias from the previous evaluations. The MRI images could be freely adjusted in all multiplanar reconstructions for optimal visualization of the scaphoid bone.

The analysis included the assessment of scaphoid fractures, stability as displayed for category A1 and A2 versus instability as displayed for category B1-4 by Schmitt and Rosenthal [2] after Herbert and Fischer [20] and Krimmer, Schmitt and Herbert [3]. For both criteria, diagnostic confidence was graded on a five-point Likert scale (1 = excellent, 5 = very poor). The need for additional imaging to reliably diagnose a scaphoid fracture was also assessed by each reader.

### Statistics

For categorical data, the absolute number and relative frequencies are presented. For ordinal data the median and interquartile range (IQR), otherwise the mean and standard deviation (SD) are presented.

Diagnostic accuracy was evaluated for X-ray and the novel 3D low-field MRI sequence in comparison with the reference standard using plots of receiver operating characteristic curves (ROC) with area under the curve (AUC) values for each reader regarding scaphoid fractures. ROC curves were calculated based on the 5-point diagnostic confidence Likert scale (1 = excellent, 5 = very poor). Due to the binary decision rule (fracture/no fracture) this resulted in a 10-point scale (1 = no scaphoid fracture with excellent certainty up to 10 = scaphoid fracture with excellent certainty). 95% confidence intervals and p-values were derived by the method of DeLong, DeLong and Clarke-Pearson [21]. In addition, for each reader, sensitivity, specificity, PPV, and NPV were calculated for the detection of the scaphoid fractures for both modalities. 95% confidence intervals were determined by a mixed logistic regression model with modality, reader and the interaction of modality and reader as fixed effects. In case sensitivity, specificity, PPV or NPV was 100%, the lower limit of the 95% confidence interval was determined using the Wilson confidence interval. Due to limited sample size and convergence issues with the mixed logistic regression model when point estimates equal 1, p-values were obtained using the exact McNemar test (sensitivity/specificity) and the Leisenring et al. (2000) method (PPV/NPV) to compare the performance of X-ray versus 3D MRI regarding scaphoid fracture detection for each reader [22]. Regarding fracture stability, sensitivity and specificity were calculated despite the small cohort.

Fleiss’ *κ* was used to evaluate inter-rater reliability. A kappa (*κ*) value of 0.00 was interpreted as no, ≤ 0.20 slight, 0.20 ≤ 0.40 fair, 0.40 ≤ 0.60 moderate, 0.60 ≤ 0.80 substantial, and 0.80 ≤ 1.00 as almost perfect agreement [23].

All *p*-values should be interpreted descriptively. No adjustment for multiplicity was done.

The statistical analysis was performed with SAS® v9.4 and R v4.5.0.

## Results

**Fig. 2:**
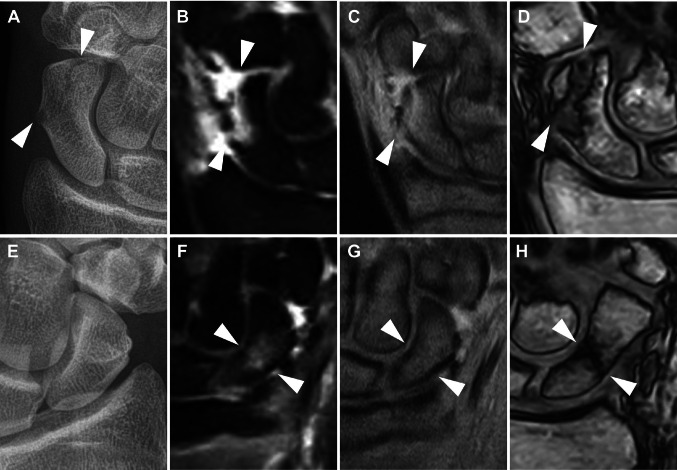
X-ray (**A**, **E**), standard MRI with coronal STIR (**B**, **F**) and coronal T1 in-phase (**C**, **G**), and the high-resolution 3D T1-weighted MRI sequence (**D**, **H**) of a stable scaphoid fracture of the tubercle type A1 in a 33-year-old female patient (**A**-**D**) and a non-displaced transverse fracture of the waist type A2 in a 28-year-old male patient (**E**–**H**). All fractures (indicated by arrowheads) are clearly detectable in the 3D T1-weighted sequence whilst the transverse fracture is not visible in X-ray (**E**) and T1 in-phase imaging (**G**) and with only edema in the STIR (F)

**Fig. 3:**
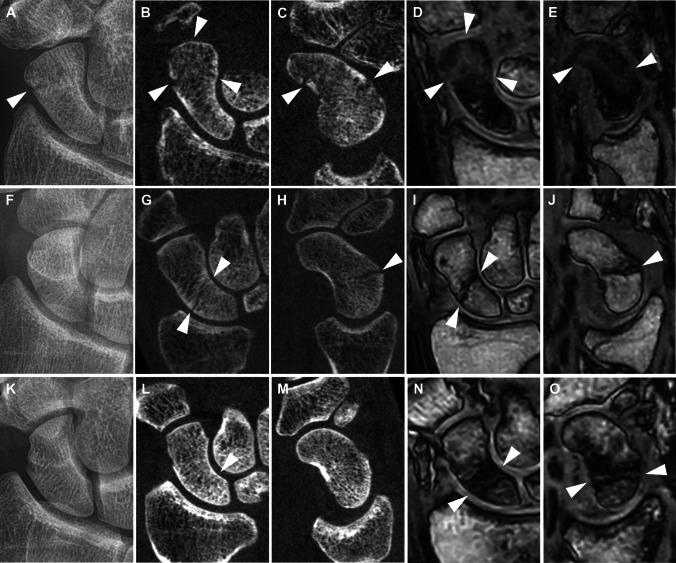
X-ray (A, F, K), CT (B/C, G/H, L/M), and high-resolution 3D T1-weighted MRI (D/E, I/J, N/O) of unstable scaphoid fractures. An extended oblique B1 type fracture in a 28-year-old male patient is shown in panels **A**-**E**, a slightly displaced transverse fracture type B2 in a 45-year-old male patient in **F**-**J**, and a fracture of the proximal third type B3 in a 37-year-old male patient in images **K**–**O**. Again, all fractures (indicated by arrowheads) are clearly detectable in the 3D T1-weighted sequence, in the first case accompanied by a marked edema (D/E). In contrast, the type B2 and B3 fractures are not visualized by X-ray (F/K) because radiographs were suboptimal due to pain. On CT, only a limited portion of the type B1 fracture is visible (G/H), whereas the type B3 fracture is barely discernible (L/M).In CT imaging the type B1 fracture is only partially visible (G/H), the type B3 fracture barely visible (L/M).

We enrolled 40 participants (mean age 38 ± 16 years; 26 men). Patient characteristics are provided in Table 2. According to the standard reference based on CT and standard MRI, 21 participants presented with fractures (53%). In 26 cases the right carpus, in 14 cases the left carpus was investigated. Fractures included different carpal and metacarpal bones without the scaphoid in 10/21 cases, the scaphoid and carpal bones in 5/21 cases and the scaphoid alone in another 6/21 cases. Therefore in 29 participants no scaphoid fracture could be observed and in 19 participants no fracture at all. Examples of scaphoid fractures are given in Figs. 2, and 3.

**Table 2:** Patient characteristics

Patient characteristics	Value
Number of patients enrolled	40
Mean age	38 years ± 16 years SD
Male sex (%)	26 (65%)
Side investigated: right/left	26/14
No fracture (%)	19 (48%)
Fracture (%)/male sex (%)	21 (53%)/16 (76%)
Scaphoid fracture	11 (28%)
A1 classification (stable)	1
A2 classification (stable)	5
B1 classification (unstable)	1
B2 classification (unstable)	3
B3 classification (unstable)	1
B4 classification (unstable)	0
Side of fracture: right/left	5/6
including other carpal or metacarpal fractures	5
Other wrist fractures without scaphoid fracture	10

### Diagnostic accuracy of the 3D T1-weighted MRI

**Fig. 4:**
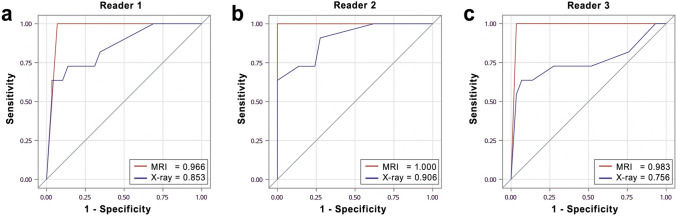
Receiver operating characteristic curves for the detection of scaphoid fractures with the 3D MRI sequence for reader 1 (**a**), 2 (**b**), and 3 (**c**)

The AUC for detection of scaphoid fractures was 0.966 (95% CI 0.919–1)/1.000 (95% CI 1–1)/0.983 (95% CI 0.949–1) for the 3D MRI sequence and 0.853 (95% CI 0.717–0.988)/0.906 (95% CI 0.804–1)/0.756 (95% CI 0.543–0.969) for X-ray for reader 1/2/3 (Fig. 4). There was a consistent numerical difference in favor of the 3D MRI sequence with an AUC-difference of 0.113 (95% CI −0.029–0.255, *p* = 0.120)/0.094 (95% CI −0.008–0.196, *p* = 0.072)/0.227 (95% CI 0.010–0.445, *p* = 0.040) for reader 1/2/3.

Averaged, the AUC was 1 (95% CI 1–1) for 3D MRI and 0.823 (95% CI 0.641–1) for X-ray (AUC-difference of 0.177, 95% CI: −0.004–0.359, *p* = 0.056).

**Fig. 5:**
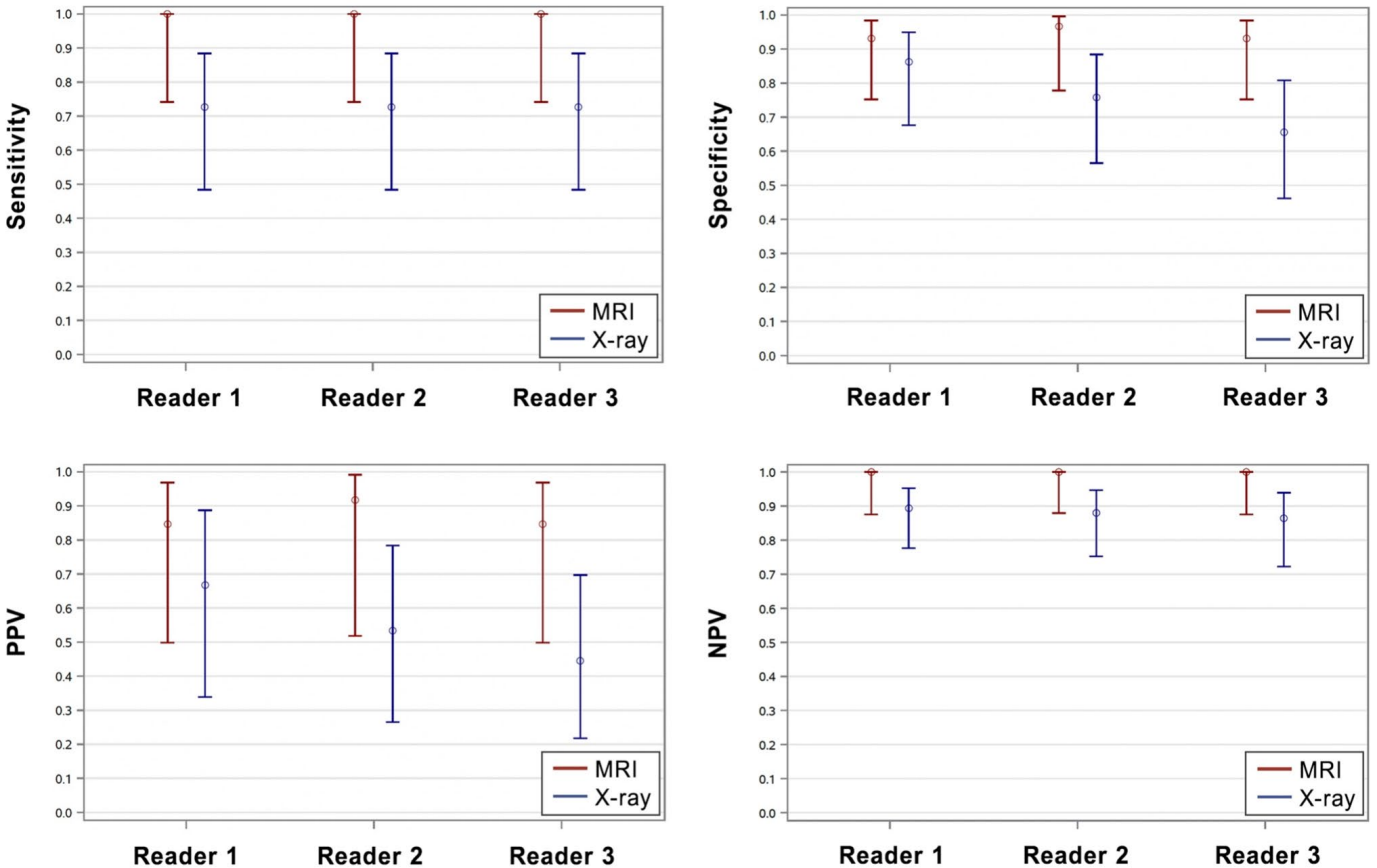
Sensitivity, specificity, PPV, and NPV with 95% confidence intervals for the detection of scaphoid fractures with the 3D MRI sequence for reader 1, 2, and 3

Point estimates of sensitivity, specificity, PPV, and NPV were higher for the 3D MRI for each reader (minimal difference of 0.069 at specificity for reader 1 [0.9310 vs 0.8621]). Sensitivity for scaphoid fracture detection was 1.000 (95% CI 0.723–1.000) for the 3D MRI sequence and 0.727 (95% CI 0.483–0.884) for X-ray for all readers (*p* = 0.250), specificity 0.931 (95% CI 0.752–0.984)/0.966 (95% CI 0.779–0.996)/0.931 (95% CI 0.752–0.984) for the 3D MRI sequence versus 0.862 (95% CI 0.676–0.949)/0.759 (95% CI 0.565–0.884)/0.655 (95% CI 0.461–0.808) for X-ray for reader 1/2/3 (*p* = 0.625/0.070/0.039). PPV equaled 0.846 (95% CI 0.498–0.968)/0.917 (95% CI 0.518–0.991)/0.846 (95% CI 0.498–0.968) for the 3D MRI sequence versus 0.667 (95% CI 0.338–0.887)/0.533 (95% CI 0.265–0.784)/0.444 (95% CI 0.218–0.697) for X-ray (*p* = 0.163/0.008/0.004), and NPV equaled 1.000 (95% CI 0.875–1.000)/1.000 (95% CI 0.879–1.000)/1.000 (95% CI 0.875–1.000) for the 3D MRI sequence versus 0.893 (95% CI 0.776–0.952)/0.880 (95% CI 0.752–0.947)/0.864 (95% CI 0.722–0.939) for X-ray (*p* = 0.068/0.068/0.067) as shown in Fig. 5.

Displacement of scaphoid fractures was presumed in case of a fracture gap > 1 mm. All fractures were correctly identified by MRI and they were correctly reported as displaced with the 3D MRI sequence in 100%/100%/33%. If detected at all with X-ray, displacement was correctly described here in only 50%/75%/25% (reader 1/2/3).

Scaphoid fractures were classified as stable (classification A1 and A2, see Fig. 2) or unstable (classification B1-B4, see Fig. 3). Five fractures were rated as unstable by the reference standard and correctly identified in 100% with the 3D MRI sequence by each reader, with X-ray in only 60%/40%/40% by reader 1/2/3. The six stable fractures were correctly identified with the 3D MRI in 3/4/3 cases and with X-ray in 2/3/4 (reader 1/2/3). X-ray only overrated two stable fracture as unstable and did not detect unstable fractures in the remaining cases. The 3D MRI detected all fractures but overrated stable fractures as unstable in 50%/33%/50% (reader 1/2/3), but altogether for detection of instability it provided a perfect low FNR of 0 and perfect sensitivity of 1 for all readers despite the lower specificity of 0.5/0.667/0.5 for reader 1/2/3 in this small collective.

### Diagnostic confidence of findings

For the 3D SST1 MRI sequence, the diagnostic confidence was rated as excellent for scaphoid fracture detection (reader 1/2/3: median 1/1/1, IQR 1–1/1–2/1–1) and for displacement of scaphoid fracture (reader 1/2/3: median 1/1/1, IQR 1–2/1–1/1–2), and excellent to moderate for stability judgement according to classification of scaphoid fractures (reader 1/2/3: median 3/2/1, IQR 2–3/1–2/1–2). Additionally, the need for more imaging to reliably diagnose or exclude a scaphoid fracture was denied in most cases (for reader 1/2/3 in 40/36/37 out of 40 cases).

In X-ray, the diagnostic confidence was rated as moderate for scaphoid fracture detection (reader 1/2/3: median 2/3/3, IQR 1–3/2–4/2–4), excellent to moderate for displacement of scaphoid fracture (reader 1/2/3: median 3/2/1, IQR 1.75–3/1–2.5/1–1) and stability judgement according to classification of scaphoid fractures (reader 1/2/3: median 1/3/3, IQR 1–2/2–3/2–3.75). The need for more imaging after X-ray was denied in fewer cases than after the single MRI sequence (for reader 1/2/3 in 27/8/13 out of 40 cases).

### Inter-rater reliability

Inter-rater reliability was nearly perfect for scaphoid fracture detection (0.961, 95% CI 0.888, 1.000, *p* < 0.001) in the 3D MRI sequence, it was moderate for scaphoid fracture detection (0.502, 95% CI 0.288, 0.716, *p* < 0.001) in X-ray.

## Discussion

This prospective study evaluated the diagnostic accuracy of a single high-resolution T1-weighted 3D gradient-echo sequence in low-field MRI for scaphoid fracture detection. In the pilot cohort, the sequence achieved a high diagnostic accuracy, with perfect sensitivity and negative predictive value in this dataset, a high specificity, and a very good overall discrimination. Across readers, diagnostic performance was consistently numerically higher than for radiography, and reader confidence and inter-rater agreement were high.

We also exploratorily assessed scaphoid fracture stability. Because only five unstable fractures were present, these findings should be interpreted cautiously and as hypothesis-generating only. Although sensitivity for instability was high in this dataset, the 3D sequence tended to overcall instability, resulting in limited specificity, which might be owed to artifacts particularly along or close to the scaphoid cortex.

To increase the diagnostic yield of the initially performed radiographs in patients with wrist trauma, X-ray may be repeated after one or two weeks when an occult scaphoid fracture is suspected. This approach may detect additional fractures, but it can delay appropriate treatment or lead to unnecessary immobilization [24]. Recent meta-analyses report that deep-learning algorithms detect scaphoid fractures on radiographs with pooled AUCs of 0.77–0.96, sensitivity only around 0.80 and specificity around 0.89, values comparable to experienced clinicians [25]. These findings indicate that AI applied to radiographs misses a proportion of scaphoid occult fractures as well. Therefore, further or alternative imaging is required, which most commonly implies carpal CT or MRI. Nonetheless, CT is associated with additional radiation exposure. MRI, on the other hand is radiation-free and highly sensitive for assessing carpal fractures including scaphoid fractures [2, 12–16]. For 3 T MRI, a 3D T1-weighted GRE CT-like sequence has been established and has shown convincing performance for fracture detection, with excellent sensitivity, specificity and accuracy, and high image quality acquired in less than 4:30 min [19]. Another study confirmed high performance of different CT-like MRI sequences, especially a gradient-echo sequence with radial k-space filling, for accurate assessment of bone morphology using a 3 T MRI [26]. However, 3 T MRI are not available in all settings. Although low-field MRI systems generally have limitations in image quality such as lower resolution, they may be designed for several special purposes and more easily available in acute trauma situations [27]. Previous studies already confirmed the superiority of dedicated musculoskeletal low-field 0.1 T MRIs over radiographs in case of occult fractures [28, 29]. Our study likewise shows promising results for using an approximately 4-min 3D GRE sequence on a dedicated musculoskeletal low-field MRI of 0.31 T. Due to the use of an extremely short repetition time with a spoiler radiofrequency and low flip angle a T1 contrast is acquired. At bone cortices or cortical discontinuities such as fractures, a pronounced hypointense signal is occurring due to boundary artifacts. This may contribute to the high sensitivity and negative predictive value observed for this 3D sequence. These striking characteristics make MRI and such sequences a promising and fast screening tool. However, low-field MRI scanners are not available everywhere and have a limited range of applications. Due to the relatively low effort required to operate those devices as well as the lower spatial and financial demands, and their particular focus on the musculoskeletal system, they appear to be especially interesting for emergency settings. A comparison of these devices and high-resolution sequences across different MRI scanners would be useful in order to evaluate the potential of such rapid screening sequences and possibly make them accessible across multiple device options and clinical settings. This might be of particular interest as cost analyses revealed that early MRI in patients with suspected occult scaphoid fractures can be cost-saving while improving diagnostic workflow and patient satisfaction [30, 31].

Cross-sectional imaging with CT or MRI remains necessary when radiographs are negative but clinical suspicion for occult scaphoid fracture persists [32]. To further reduce costs and avoid delay of early treatment, and thereby potentially lowering the risk of fracture-related complications, initial imaging in patients with wrist trauma and suspected fracture could also be performed using a very brief MRI protocol, where available.

A major limitation of our study is the small sample size and the limited number of scaphoid fractures, which reduces the precision of the reported diagnostic performance estimates and the robustness of the inter-reader agreement analysis. In particular, fracture classification and stability assessment should be interpreted cautiously and as exploratory only, because only five unstable fractures were present. Moreover, participants were recruited from a preselected single-center cohort of patients with clinical suspicion of a scaphoid fracture and a clinical indication for wrist CT. This likely contributed to the high fracture prevalence in our cohort and may have inflated diagnostic performance estimates, thereby limiting generalizability to unselected patients with suspected scaphoid fracture. Finally, the study was performed on a single dedicated 0.31-T extremity MRI system and evaluated only one 3D T1-weighted sequence. Transferability to other low-field platforms or to routine 1.5/3 T MRI remains uncertain, and isolated bone bruises or trabecular injuries may be missed without additional edema-sensitive sequences.

Overall, the single 3D SST1 sequence on a dedicated low-field MRI scanner showed promising diagnostic performance for scaphoid fracture detection in this prospective pilot cohort, with perfect sensitivity and NPV in this dataset and high reader confidence. These preliminary findings support its potential role as a rapid screening tool within a low-field wrist MRI workflow, but confirmation in larger and less selected cohorts is required.

## Data Availability

Data are available from the corresponding author on reasonable request.

## Abbreviations

AUC: Area under curve
CI: Confidence interval
CT: Computed tomography
FNR: False negative rate
IQR: Interquartile range
MRI: Magnetic resonance imaging
NPV: Negative predictive value
PPV: Positive predictive value
ROC: Receiver operating characteristic curves

## Declarations

### Trial registration

The trial was prospectively registered at the German Clinical Trials Register (DRKS00014256, registration date 13/03/2018).
